# Response of Functional Traits of Aquatic Plants to Water Depth Changes under Short-Term Eutrophic Clear-Water Conditions: A Mesocosm Study

**DOI:** 10.3390/plants13101310

**Published:** 2024-05-09

**Authors:** Yang Liu, Leah Ndirangu, Wei Li, Junfeng Pan, Yu Cao, Erik Jeppesen

**Affiliations:** 1 Research Center of Aquatic Plant, Wuhan Botanical Garden, Chinese Academy of Sciences, Wuhan 430074, China; liuyang1025future@163.com (Y.L.); ndiranguleah@gmail.com (L.N.); liwei@wbgcas.cn (W.L.); caoyu@wbgcas.cn (Y.C.); 2 Hubei Key Laboratory of Wetland Evolution & Ecological Restoration, Wuhan Botanical Garden, Chinese Academy of Sciences, Wuhan 430074, China; 3 Yani Wetland Ecosystem Positioning Observation and Research Station, Tibet University, Lhasa 850000, China; 4 Key Laboratory of Biodiversity and Environment on the Qinghai-Tibetan Plateau, Ministry of Education, Tibet University, Lhasa 850000, China; 5 Horticulture and Conservation Centre, Wuhan Botanical Garden, Chinese Academy of Sciences, Wuhan 430074, China; 6 Department of Ecoscience, Aarhus University, 8000 Aarhus, Denmark; ej@ecos.au.dk; 7 Limnology Laboratory, Department of Biological Sciences and Centre for Ecosystem Research and Implementation, Middle East Technical University, Üniversiteler Mahallesi, Çankaya, Ankara 06800, Turkey; 8 Sino-Danish Centre for Education and Research (SDC), Beijing 100049, China; 9 Institute for Ecological Research and Pollution Control of Plateau Lakes, School of Ecology and Environmental Science, Yunnan University, Kunming 650032, China

**Keywords:** aquatic plant, water depth, functional traits, eutrophic clear water

## Abstract

Aquatic plants play a key role in the structuring and functioning of shallow lake ecosystems. However, eutrophication often triggers shifts in plant communities and species diversity, especially in the early stages when the water is still clear. Additionally, water depth is an important factor regulating aquatic plant communities. We conducted a 50-day mesocosm study to investigate how water depth (50 cm and 100 cm) affected the functional traits (vertical expansion versus horizontal colonisation) of 20 aquatic plants under eutrophic clear-water conditions. Among the selected species, the submerged plants *Hydrocotyle vulgaris* and *Limnophila indica* exhibited higher plant height or biomass in deeper water, while the emergent plants *Myriophyllum aquaticum* showed the opposite trend. Additionally, *Ludwigia peploides* subsp. *stipulacea* exhibited better vertical growth than the remaining species, and the submerged species *Vallisneria denseserrulata* had better horizontal colonisation. There was a positive correlation between plant height and rhizome length, indicating the absence of a trade-off between vertical growth and horizontal expansion. Our findings suggest an overall resilience of aquatic plants to varying water depths within our study range and highlight the importance of analysing functional traits when selecting appropriate species in freshwater ecosystem restoration, particularly in the face of climate change-induced water depth fluctuations.

## 1. Introduction

Aquatic plants are important primary producers in shallow lakes and play a key structuring role in such systems [1]. The plants can facilitate clear-water conditions by absorbing excessive nutrients and by limiting, both directly and indirectly, phytoplankton growth [2]. Furthermore, aquatic plants can support macroinvertebrate, fish, and periphyton growth by providing heterogeneous habitats and substrates [1]. However, aquatic plant communities have experienced a significant decline in recent decades [3,4], and some macrophyte species have become threatened or extinct [5,6,7]. Multiple factors have contributed to the decline in macrophytes and specific species changes, including changes in climate [8], heavy metal pollution [9], invasions [10], cyanotoxins [11], and, in particular, eutrophication [12].

Eutrophication is usually characterised by a turbid water state with phytoplankton dominance [13]. Previous studies have shown major shifts in aquatic plant communities with eutrophication [14,15]. As an example, communities dominated by charophytes or *Vallisneria* spp. have shifted to dominance by *Myriophyllum spicatum* or *Stuckenia pectinata* in the middle and lower reaches of the Yangtze River and the lakes of the Yunnan–Guizhou Plateau [15]. However, in the early stage of eutrophication, the water may remain clear even when nutrient levels are increasing [13]. Aquatic plants are usually strongly limited by light even in the early period of eutrophication when the water is still clear, as a result of a rapid increase in periphyton abundance leading to shading [6,16,17,18]. In some cases, however, the increased nutrient availability has counteracted the negative effects of periphyton shading, leading to increased plant growth despite the higher periphyton biomasses [19].

As light attenuates strongly through the water column, water depth may be of vital importance in regulating macrophyte communities, not least under eutrophic conditions [20]. Previous studies have investigated the functional traits of single species (e.g., *Potamogeton maackianus*) or the mechanisms ruling species assemblies in aquatic communities along water depth gradients in natural lakes [21,22]. However, the effects of water depth were hard to disentangle in these studies as they were conducted in natural lakes influenced by other factors such as wind, fish, and sediment type [23,24,25], which also affect the growth of aquatic plants. 

Functional traits are a series of core properties closely related to the survival, growth, and reproduction of plants [26]. The trait-based leaf–height–seed approach has been applied to understand the growth and distribution pattern of plants along environmental gradients, and, at a global scale, functional traits have been considered vital for analysing the species composition of prevailing plant communities [27,28,29]. For example, a global-scale survey based on more than 2000 species revealed a typical trade-off between different functional traits (e.g., photosynthesis rate and leaf life span) of plant leaves [29]. In addition, functional-trait approaches have been broadly applied to optimise restoration practices for plant re-establishment of terrestrial ecosystems [30,31], but less so for aquatic ecosystems. In our study, the functional traits of 20 aquatic plants and their responses to two levels of water depth under eutrophic clear-water conditions were investigated in mesocosms. The traits included morphological indicators (e.g., plant height and biomass). We aimed to elucidate (1) the effects of water depth on the functional traits of aquatic plants and (2) the trade-offs of functional traits between the vertical expansion and horizontal colonisation of the selected species. We further aimed to identify suitable species for the restoration of shallow aquatic ecosystems based on plant functional traits. We hypothesised that greater depth could inhibit the horizontal expansion of aquatic plants and promote vertical growth to compensate for reduced light availability, and trade-offs between the traits of vertical growth and horizontal expansion are therefor expected. Our study provides insight into the dynamics of aquatic plant communities in the early phase of eutrophication when the lakes are still in a clear-water state.

## 2. Results

### 2.1. Water Physico-Chemical Parameters, Periphyton Biomass, and Phytoplankton Biomass

The environmental variables are listed in Table 1. Phytoplankton chlorophyll a (Chla) was low during the experiment. In addition, PAR (photosynthetically active radiation) at the water surface was ca. 300–1000 µmol m^−2^ s^−1^ at midday, and Kd showed a ca. 33% reduction in PAR at 50 cm and a ca. 50% reduction at 100 cm.

**Figure 1:**
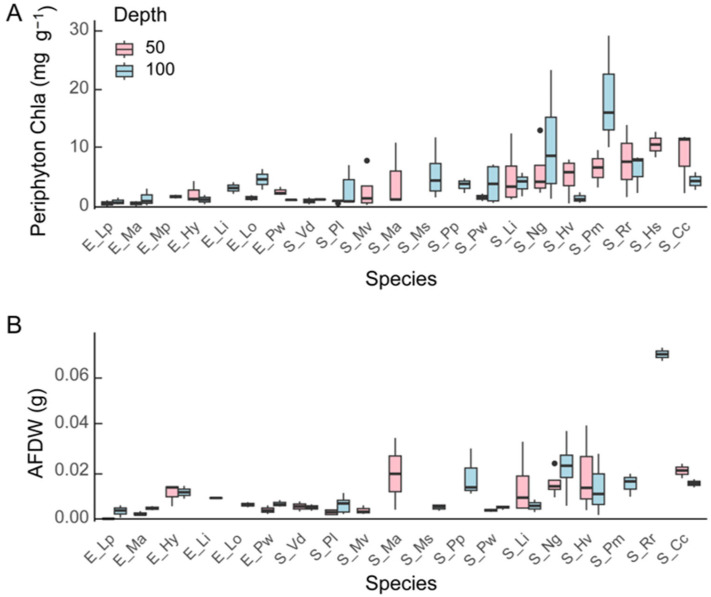
Boxplots of periphyton Chla (**A**) and ash free dry weight (AFDW, (**B**)) from macrophytes at 50 cm and 100 cm depth (*n* = 3–5). ‘E’ refers to emergent macrophytes; ‘S’ refers to submerged macrophytes. The species listed in the figure are indicated by the two initial letters of their species name. Lp stands for *L. peploides* subsp. *stipulacea*, Ma for *M*. *aquaticum*, Hy for *H*. *vulgaris*, Li for *L*. *indica*, Lo for *L*. *ovalis*, Pw for *P*. *wrightii*, Vd for *V*. *denseserrulata,* Pl for *P. lucens*, Mv for *M. verticicillatum*, Ms for *M. spicatum*, Pp for *P. perfoliatu*, Ls for *L. sessiliflora*, Mp for *M. propinquum*, Hs for *H*. *salicifolia*, Hv for *H*. *verticillata*, Ng for *N*. *guadalupensis*, Pm for *P. maackianus*, Po for *P. octandrus*, Rr for *R*. *rotundifolia,* and Cc for *C*. *caroliniana*. The middle line of the box plot represents the median; the upper and lower bars indicate the third quartile and first quartile, respectively. Dots outside the boxplot are outliers.

The two indicators of periphyton biomass (Chla and AFDW) exhibited no significant differences between the two water depths using a Mann–Whitney U test for each species (Figure 1, Table S2). However, at 100 cm depth, periphyton on *P. maackianus* (S_Pm) had a higher Chla content (18.5 ± 9.7 mg g^−1^) and that of *R. rotundifolia* (S_Rr) had a higher AFDW content (0.092 ± 0.068 mg g^−1^) than the other species.

### 2.2. Effects of Water Depth on Plant Function Traits

Only a few traits and species showed significant differences between the two water depths (50 and 100 cm) for the functional traits (height, leaf length, biomass, etc.) of the plants (Table 2). The height of emergent *M. aquaticum* (E_Ma) differed significantly, being two times higher at 50 cm than at 100 cm, while the height of *H. verticillata* (Hv) was three times higher at 100 cm than at 50 cm.

The leaf length of emergent *M. aquaticum* responded markedly to water depth, whereas the remaining 19 species exhibited no significant differences. The leaf length of emergent *M. aquaticum* was 3.11 ± 0.14 cm at 50 cm and 0.87 ± 0.09 cm at 100 cm (Table 2).

The biomass of the submerged *L. indica* (S_Li) and the emergent *M. aquaticum* as well as *L. peploides* subsp*. stipulacea* (E_Lp) responded significantly to the changes in water depth. The biomass of submerged *L. indica* at 100 cm was three times higher than that at 50 cm, while the biomass of *L. peploides* subsp*. stipulacea* was about three times higher at 50 cm than at 100 cm, and that for emergent *M. aquaticum* was almost 20 times higher at 50 cm than at 100 cm.

No differences in ramet number and rhizome length were found between the two water depths for any of the studied species.

### 2.3. Species Variation of Functional Traits in 20 Aquatic Plants

**Figure 2:**
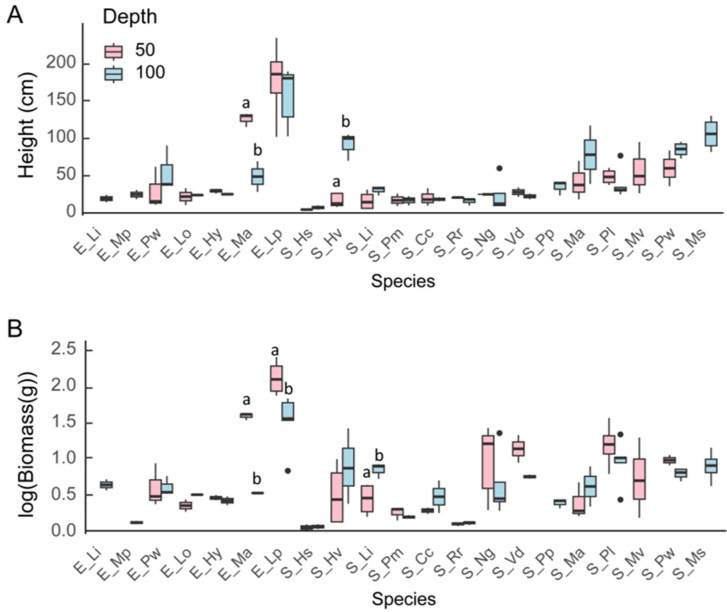
Boxplots of plant height (**A**) and biomass (**B**) of different macrophytes at 50 cm and 100 cm depth (*n* = 3~5). Boxplots show median, inter-quartile range, and minimum and maximum values. “a” and “b” refer to statical significance. The middle line of the box plot represents the median; the upper and lower bars indicate the third quartile and first quartile, respectively. Dots outside the boxplot are outliers.

*L. peploides* subsp*. stipulacea* was the longest plant at the end of the experiment at both depths, and emergent *M. aquaticum* was second longest at the shallow water depth (Figure 2). Furthermore, *L. peploides* subsp*. stipulacea* had the largest biomass of all species, with a maximum of 149 ± 84 g at 50 cm depth (Figure 2).

**Figure 3:**
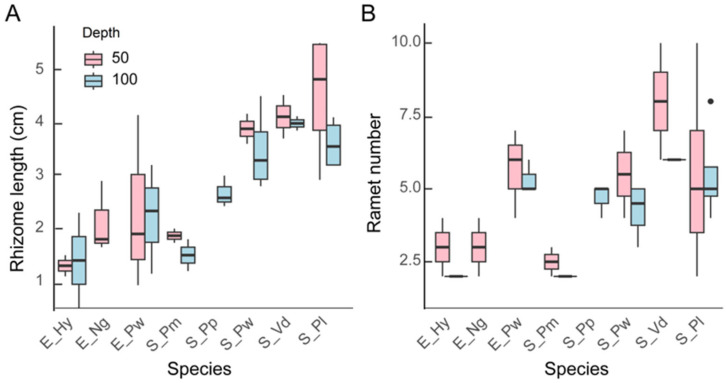
Boxplots of rhizome length (**A**) and ramet number (**B**) of different macrophytes at 50 cm and 100 cm depth (*n* = 3~5). The description of the box whiskers is explained in Figure 1.

The ramet number did not differ significantly among the species at 50 cm, while at 100 cm, *H. vulgaris* (E_Hy) and *P. macckianus* (S_Pm) had a significantly smaller ramet number than the emergent *P. wrightii* (E_Pw), *P. lucens* (S_Pl), and *V. denseserrulata* (S_Vd) (Figure 3). The rhizome length of *H. vulgaris* was shorter than that of *P. lucens* at 50 cm and of *V. denseserrulata* at 100 cm (Figure 3). The maximum recorded rhizome length was 4.5 ± 1.2 cm (*P. lucens* at 50 cm depth).

No significant difference in ramet traits was observed between the two depths when using a Mann–Whitney U test for each species.

### 2.4. Relationship between Functional Traits of Vertical Growth and Horizontal Expansion

**Figure 4:**
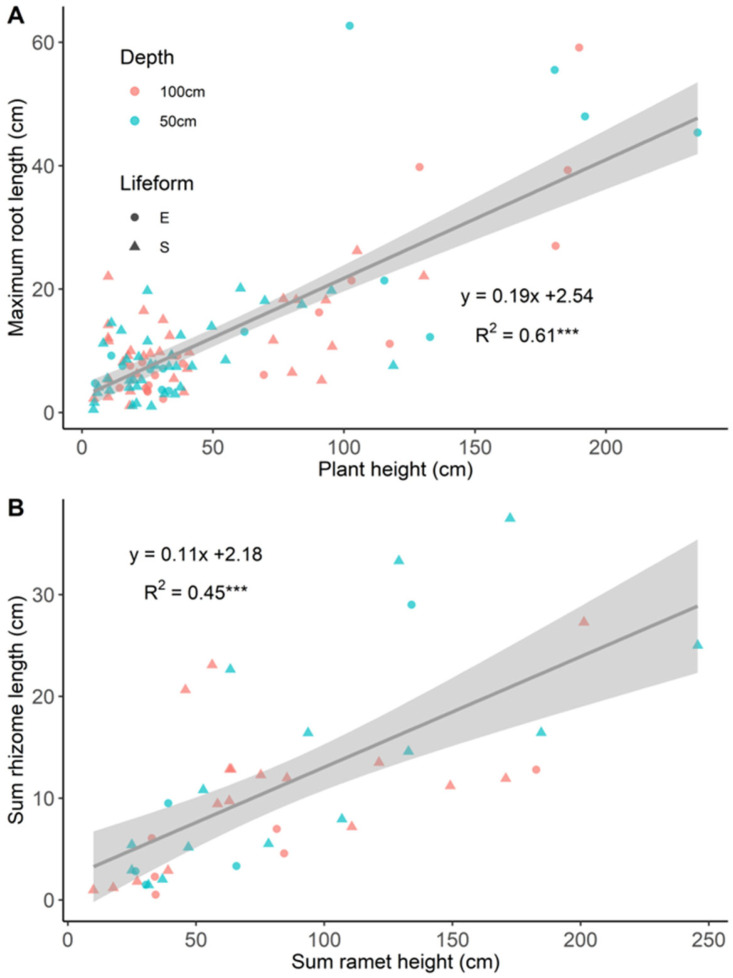
Scatterplots of plant height and maximum root length (**A**), vertical growth (sum of ramet height) and horizontal colonisation (sum of rhizome length) (**B**) at 50 and 100 cm. *** *p* < 0.001. Green and orange refer to the 50 cm and 100 cm depth results, respectively. ‘●’ and ‘▲’ indicate emergent and submerged forms, respectively.

Plant height was positively related to maximum rhizome length (R^2^ = 0.25, *p* < 0.001). Similarly, a significant relationship was found between plant height and ramet numbers. Moreover, the higher the plants, the longer their roots (R^2^ = 0.61, *p* < 0.001) (Figure 4A); for instance, *L. peploides* subsp. *stipulacea* had the largest plant height (193.9 ± 20.7 cm) and root length (45.7 ± 11.6 cm).

In addition, we found that the sum of the ramet height correlated well with the sum of the rhizome length (R^2^ = 0.45, *p* < 0.001) (Figure 4B). However, no correlation was recorded between the average ramet height and ramet number (R^2^ < 0.01, *p* > 0.05).

### 2.5. Relationship between Aquatic Plants and Periphyton Traits

**Figure 5:**
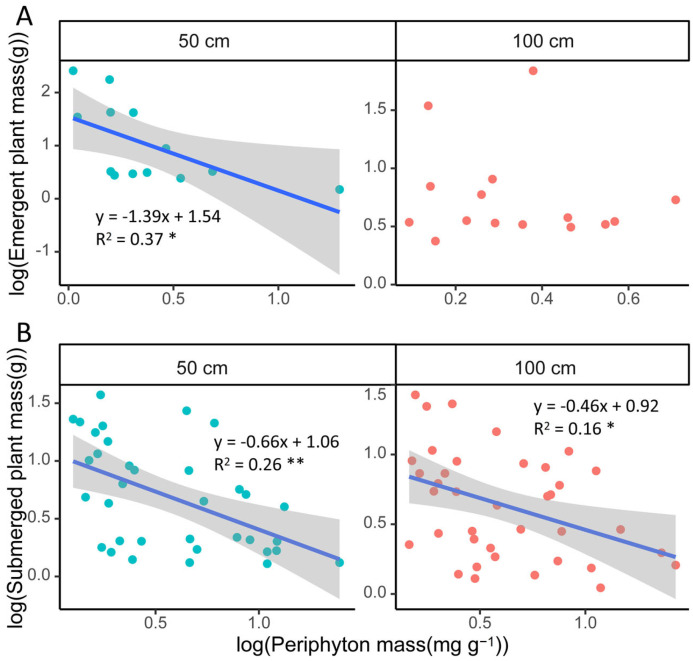
Scatter plots for the biomass of periphyton and emergent macrophytes (**A**) and submerged macrophytes (**B**) at 50 cm and 100 cm depth. Periphyton mass refers to periphyton Chla per macrophyte biomass. The axes were log-10 transformed. Lines fitted in the plots represent relationships between periphyton biomass and macrophyte biomass. * *p* < 0.05, ** *p* < 0.01. Green and orange refer to the 50 cm and 100 cm depth results, respectively.

The biomass of emergent macrophyte species did not correlate significantly with periphyton biomass at 100 cm depth, whereas it was negatively correlated with periphyton Chla and AFDW at 50 cm. However, the biomass of submerged macrophytes was negatively correlated with periphyton biomass at both depths (Figure 5, Table S4).

## 3. Discussion

### 3.1. Water Depth Impacts on Plant Traits

Three species, *H. verticillata* (plant height), *L. indica* (biomass), *L. peploides* subsp. *Stipulacea* (biomass) and *M. aquaticum* (plant height, leaf length, and biomass), were affected by water depth. Correspondingly, Wei et al. [32] found that the optimal water depth for *H. verticillata* growth within the range of 30–150 cm was 90 cm under favourable light conditions. *M. aquaticum* has optimum growth in wetlands and shallow water [33], which supports our finding that emergent *M. aquaticum* had a larger size, including plant height, leaf length, and biomass, at 50 cm than at 100 cm. However, water depth did not have significant impacts on plant traits for most of the selected species, which contrasts the findings of previous studies demonstrating significant effects of water depth on plant biomass, plant height, and ramet size [20,34,35,36]. This probably reflects the modest shading from phytoplankton in our study, leading to relatively low light attenuation in the water throughout the experiment. In our study, Kd (1.3 m^−1^) was low compared with the Kd (3.1 m^−1^) recorded by Wang et al. [37], who found a significant decrease in the biomass and plant height of *P. perfoliatus* and *M. spicatum* when water depth increased from 30 cm to 150 cm. Consistent with our results, Middelboe and Markager [38] found insignificant responses of the plant height of aquatic angiosperms when the water depth increased from 0.5 m to 1.5 m in clear-water lakes with Kd < 1 m^−1^. Our results are therefore relevant from a climate change perspective as they show plants to be resilient to moderate variations in water depth (e.g., induced by extreme climate events) as long as the water stays clear.

Although the periphyton biomass recorded in our study was higher than those of previous studies [39,40], no significant differences in periphyton biomass occurred between the two depths. And periphyton biomass showed a negative correlation with the biomass of submerged macrophytes at both depths, suggesting that periphyton shading plays a significant role for submerged macrophytes, though less so for emergent macrophytes [41].

### 3.2. Trade-Offs between Plant Functional Traits

Vertical growth was positively related to the horizontal expansion, and no trade-offs were revealed between the traits of vertical growth and horizontal expansion in our experiment, which can be attributed to the overall shallow depths in our study. A field study has also revealed a positive relationship between vertical growth (plant biomass) and horizontal expansion (branch number) when light attenuation was only 40–60% from the water surface to 100 cm depth [42]. Under conditions of limited light availability, a potential strategy for aquatic plants could involve reallocating resources towards increased vertical growth to gain more light and then produce less [20]. Furthermore, the adequate nutrient supply (high levels of TN and TP) in our mesocosms may provide enough resources for both vertical and horizontal growth. Meanwhile, significant trade-offs between two traits are commonly discovered under constrained light or nutrient conditions [20]. Additionally, consistent with a previous study [43], there was a positive correlation between plant height and maximum root length across the aquatic species studied, implying a strong link between aboveground and belowground growth.

### 3.3. Shallow Lake Restoration with Aquatic Plants

Aquatic plant restoration plays a vital role in contemporary ecological conservation and environmental management. Aquatic plants possess the ability to filter both inorganic and organic pollutants in water, including nitrates, heavy metals, pesticides, and microplastics [44]. Functional traits are useful metrics when selecting aquatic species for the restoration of aquatic ecosystems. For littoral macrophyte re-establishment, the emergent plant *M. aquaticum*, notorious for its invasiveness [45], is commonly used in lake restoration projects in China due to its tolerance to eutrophication and other forms of pollution [44]. Since *L. peploides* subsp. *stipulacea* is a native species growing in the same niche as the invasive species, it might be a better candidate for littoral zone restoration. Our study also showed that *V. denseserrulata* had a strong ability to expand horizontally due to its large ramet number and relatively small plant height, and it therefore seems to be a good candidate for lake restoration, as also stated elsewhere [46]. Another submerged species, *P. lucens,* with abundant ramets and larger height, could be used in specific restoration projects that allow the macrophytes to reach the water surface.

Our study was conducted within mesocosms rather than in natural field conditions. It is important to note that aquatic plants in natural ponds and lakes are often exposed to waves and herbivory, which can also affect the re-establishment of aquatic plants, especially submerged macrophytes. Therefore, the outcomes of our mesocosm study should be interpreted with caution. A further combination of field experiments with our results would provide a more comprehensive understanding of aquatic plant dynamics and inform more effective management strategies.

## 4. Materials and Methods

### 4.1. Experimental Materials and Design

The experiment was conducted in the Wuhan Botanical Garden (Wuhan, China), located in a subtropical zone (30°33′ N, 114°24′ S). Macrophytes were collected from the Wuhan Botanical Garden and included 11 submerged species (*Cabomba caroliniana* A. Gray*, Myriophyllum spicatum* L., *Myriophyllum verticillatum* L., *Najas guadalupensis* (Spreng.) Magnus, *Hygrophila salicifolia* (Vahl) Nees, *Hydrilla verticillata* (L.f.) Royle*, Potamogeton lucens* L., *Potamogeton maackianus* A. Benn., *Potamogeton octandrus* Poir., *Potamogeton perfoliatus* L., and *Vallisneria denseserrulata* (Makino) Makino), five emergent species (*Hydrocotyle vulgaris* L., *Limnophila sessiliflora* (Vahl) Blume, *Ludwigia ovalis* Miq., *Ludwigia peploides* subsp*. stipulacea* (Ohwi) P.H. Raven, and *Myriophyllum propinquum* A. Cunn.), and four species with both emergent and submerged forms (*Limnophila indica* (L.) Druce*, Myriophyllum aquaticum* (Vell.) Verdc.*, Potamogeton wrightii* Morong, and *Rotala rotundifolia* (Buch.-Ham. ex Roxb.) Koehne). The species listed in this manuscript are indicated by two letters that were combined by the initial letter from the genus and the specific epithet. ‘S_’ represents submerged life form and ‘E_’ emergent life form. For example, S_Ms refers to the submerged macrophyte *M. spicatum*. Since the abbreviations of both *Hydrilla verticillata* and *Hydrocotyle vulgaris* are Hv, the latter is listed as Hy.

The mesocosm system consisted of five concrete tanks (length × width × depth: 200 cm × 200 cm × 100 cm) that were filled with water from the nearby Donghu lake (Figure S1). Each tank had four rows with plastic boxes, two being positioned at 50 cm and the other two at 100 cm depth (i.e., at the bottom of the tank), which contained all species placed at both depths. Every row had 12 plastic boxes (length × width × height: 35 cm × 9 cm × 8.3 cm), connected with iron wire. All boxes were filled with sand to a depth of about 2 cm, and commercial fertiliser was added (2.6 g, nitrogen and phosphorus content ca. 20%). The nutrient release rates of the fertiliser were calculated by a 25-day test trial from 10 October to 4 November 2017 using the same fertiliser in a 40 L bucket without macrophytes along with this study, and the rates were 8.0 mg L^−1^ of nitrogen and 1.1 mg L^−1^ of phosphorus per day (similar to 10 mg L^−1^ of nitrogen and 0.2 mg L^−1^ of phosphorus in the high nutrient treatments of González Sagrario et al. [47]). In total, based on the test trial, ca. 360 mg of nitrogen and 50 mg of phosphorus were estimated to be released from the added fertiliser. The initial TN and TP in the mesocosms were 0.83 ± 0.27 mg L^−1^ and 60 ± 6 μg L^−1^, respectively. After plant cultivation, high levels of TN and TP (TN: 5.18 ± 4.58 mg L^−1^; TP: 733 ± 327 μg L^−1^) were recorded in the water column throughout the experiment, resembling the eutrophic levels recorded in summer in heated mesocosms in an experimental setup in Denmark (TN: 5.2 mg L^−1^, TP: 0.6 mg L^−1^, Trochine et al. [48]) and another heated mesocosm experimental setup in China (TN: 6 mg L^−1^, Pacheco et al. [24]). Due to high plant coverage in the experimental setup, a clear-water state was maintained at these high nutrient levels.

For each species, apical shoots with a length of 20 cm (except for 8.5 cm for emergent *P. wrightii* and 6 cm for *Hygrophila salicifolia*) were cultivated in a separate cubicle of the box. The experiment lasted for 50 days from 7 September (Sep) to 28 October (Oct) 2017. Five tanks were used as replicates for each water depth. More information on the experimental setup is presented in Table S1 in the Supplementary Materials.

### 4.2. Sampling Methods

Water physico-chemical variables were measured monthly in each tank. Water temperature, conductivity, dissolved oxygen (DO), and the oxidation–reduction potential (ORP) were measured in situ using a YSI ProPlus multiparameter metre at 50 cm depth below the water surface in each tank. The light attenuation coefficient (Kd) was calculated based on photosynthetic active radiation at 50 cm and at the water surface using a light quantum instrument (Li-1400) following the method of Kirk [49]. One-litre water samples were collected at ca. 50 cm depth on 4 Sep, 8 Oct, and 28 Oct 2017 for water chemical analyses. To determine total phosphorus (TP) and total nitrogen (TN) concentrations, the water samples were digested with K_2_S_2_O_8_ and measured spectrophotometrically according to Lorenzen [50]. Alkalinity was determined by Gran’s titration with 0.1 mM HCl [51]. Phytoplankton chlorophyll a (Chla) was determined by filtering 500 mL of water through Whatman GF/C filters followed by extraction with 95% ethanol for 24 h, modified from Lorenzen [50].

The macrophytes were carefully harvested by the end of the experiment, and dry weight was measured after drying the plants for 48 h at 80 °C. To determine periphyton biomass, the collected macrophytes were kept in plastic bags and stored in a cool box. The macrophytes were cleaned with tap water, and the total volume of the washed-off water was recorded in the lab. A 200–500 mL sub-sample was filtered through Whatman GF/C filters and extracted by ethanol to determine the Chla content [50]; another sub-sample was likewise filtered through Whatman GF/C filters to determine the ash free dry weight (AFDW) by drying the plant material at 80 °C in an oven for 24 h, after which it was placed in a muffle furnace at 550 °C for two hours. Periphyton Chla and AFDW were calculated based on the macrophyte fresh weight.

Most plants in freshwaters maintain their species population and expand their habitat through asexual reproduction, for instance, by increasing their ramet number and rhizome length [52,53,54]. Thus, we measured morphological indicators including plant height, root length, rhizome length (the distance between two adjacent ramets), ramet number, and leaf length using a ruler for the length measurements. The sum of the ramet height and the sum of the rhizome length and average ramet height were calculated for ramet-producing plants. The maximum rhizome length was the longest rhizome for a ramet-producing plant. In general, plant height and root length represented vertical growth, and rhizome length, maximum rhizome length, and ramet number represented horizontal expansion.

### 4.3. Data Analysis

A non-parametric Mann–Whitney U test was used to analyse the effects of water depth on the plant variables (e.g., plant height, leaf length, and biomass) for each species due to violation of normal distribution. One-way ANOVA was performed to compare the difference of plant traits at each water depth. To satisfy the presumption of the ANOVA, the data were log (x + 1) transformed, if needed. The Tukey method was chosen as a post hoc test using the package ‘multcomp’ in the software R (version 3.4.2). The data are shown as mean ± SD. Pearson correlations were used to analyse the relationships between the functional traits of vertical growth and horizontal expansion, as well as between periphyton biomass and macrophyte biomass.

## 5. Conclusions

Our results show that aquatic plants are resilient to variations in water depth under clear-water conditions, which is of importance for predicting the effect of water level changes due to, e.g., extreme climate events. In addition, our findings are useful for lake managers in their selection of the most suitable aquatic plant species for ecological restoration. Among the species studied, *L. peploides* subsp*. stipulacea*, *V. denseserrulata*, and *P. lucens* emerge as preferable candidates for littoral zone restoration efforts. Since our study was conducted in mesocosms, the experimental results must be interpreted with caution, and field studies are needed in the future to verify our results.

## Figures and Tables

**Table 1:** Mean (±SD) of the physico-chemical variables in the water column during the experiment (*n* = 5), including temperature (Temp), atmospheric pressure (AP), dissolved oxygen (DO), conductivity (C), total dissolved solids (TDSs), pH, light attenuation coefficient (Kd), alkalinity (Alk), phytoplankton Chla (PhyChla), total nitrogen (TN) and total phosphorus (TP).

Sampling Day	Temp (°C)	AP mmHg	DO mg L^−1^	C µS cm^−1^	TDSs mg L^−1^	pH	Kd m^−1^	Alk mmol L^−1^	PhyChla mg L^−1^	TN mg L^−1^	TP μg L^−1^
0	25.60 ± 0.30	753 ± 0	5.72 ± 0.50	282 ± 13	207 ± 15	7.60 ± 0.05	1.20 ± 0.23	2.08 ± 0.10	14 ± 3.20	0.83 ± 0.27	60.40 ± 6.20
29	20.20 ± 0.30	761 ± 0.10	8.36 ± 1.20	331 ± 15	237 ± 11	8.60 ± 0.01	1.20 ± 0.14	1.52 ± 0.07	2.50 ± 0.30	5.18 ± 4.60	733 ± 326
50	17.80 ± 1.90	767 ± 0	12.52 ± 1.87	284 ± 15	215 ± 19	9.30 ± 0.41	1.60 ± 0.30	1.54 ± 0.23	2.80 ± 0.70	1.02 ± 0.72	91 ± 50

**Table 2:** Statistical summary of the differences in plant traits (shoot height, leaf length, and biomass) between the two depths among the 20 species using Mann–Whitney U test (*n* = 3–5). * = *p* < 0.05; NS = not significant.

Life Form	Species Name	Leaf Length		Plant Height		Biomass	
		w	Sig.	w	Sig.	w	Sig.
Emergent	*Hydrocotyle vulgaris*	7.5	NS	6	NS	4	NS
Submerged	*Cabomba caroliniana*	0	NS	3	NS	2	NS
Emergent Submerged	*Myriophyllum aquaticum*	9	*	9	*	9	*
	*Myriophyllum aquaticum*	6	NS	6	NS	6	NS
Submerged	*Hydrilla verticillata*	0	NS	0	*	3	NS
	*Najas guadalupensis*	4.5	NS	6	NS	14	NS
	*Vallisneria denseserrulata*	7	NS	3	NS	4	NS
Emergent	*Rotala rotundifolia*	3	NS	9	NS	3	NS
Emergent	*Ludwigia ovalis*	1	NS	2	NS	0	NS
	*Ludwigia peploides* subsp. *stipulacea*	5	NS	12	NS	20	*
Submerged	*Potamogeton lucens*	18	NS	16	NS	14	NS
	*Potamogeton maackianus*	4.5	NS	4	NS	6	NS
	*Potamogeton wrightii*	8	NS	2	NS	3	NS
Emergent	*Potamogeton wrightii*	12	NS	2	NS	8	NS
Submerged	*Hygrophila salicifolia*	0	NS	1	NS	1.5	NS
Emergent	*Limnophila indica*	4	NS	1	NS	0	*

## Data Availability

Data are available from the corresponding author upon reasonable request. The original contributions presented in the study are included in the article/Supplementary Materials. For additional inquiries, please reach out to the corresponding author.

## Supplementary Materials

The following supporting information can be downloaded at https://www.mdpi.com/article/10.3390/plants13101310/s1: Figure S1: Illustration of experimental preparation and tanks; Table S1: Life forms and rhizome presence/absence of the collected 20 species; Table S2: Statistical summary of the differences in periphyton Chla and AFDW between the two depths using Mann–Whitney U Test; Table S3: Statistical summary of the differences in ramet number and rhizome length between the two depths using Mann–Whitney U Test; Table S4: Correlation analysis between periphyton biomass and macrophyte biomass.
