# Supplementary material for: Response of Functional Traits of Aquatic Plants to Water Depth Changes under Short-Term Eutrophic Clear-Water Conditions: A Mesocosm Study
Source: Plants (Basel). 2024 May 9;13(10):1310. doi: 10.3390/plants13101310 (PMC11124857; doi:10.3390/plants13101310)
Supplement: Supplementary file 1 [file plants-13-01310-s001.zip › plants-2985465-supplementary.pdf]

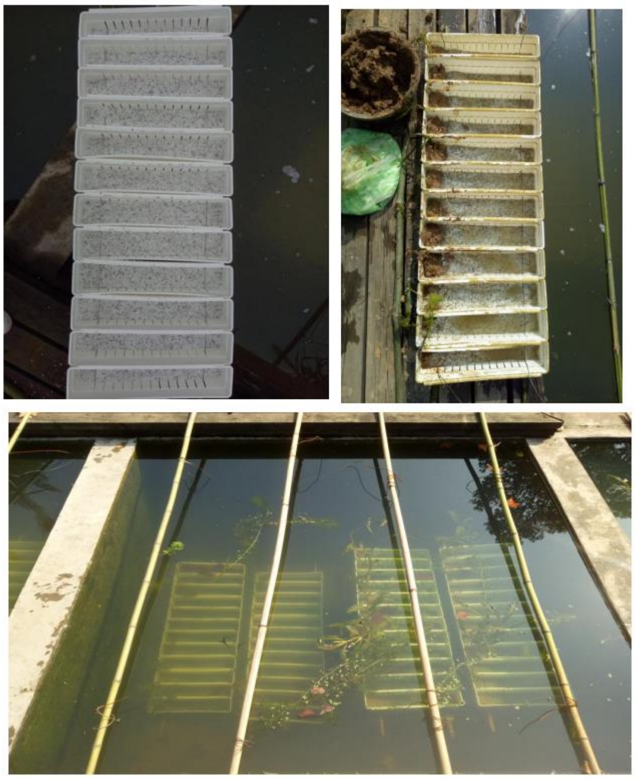

Figure S1. Illustration of experimental preparation and tanks.

Table S1. Life forms and rhizome presence/absence of the collected 20 species. "Y" and "N" mean with or without rhizomes. "E_" refers to emergent and "S_" to "submerged".

Family	Species	Abbreviation	Life form	Rhizome
Cabombaceae	*Cabomba caroliniana*	S_Cc	Submerged	N
Araliaceae	*Hydrocotyle vulgaris*	E_Hy	Emergent	Y
Ruellioideae	*Hygrophila salicifolia*	S_Hs	Submerged	N
Hydrocharitaceae	*Hydrilla verticillata*	S_Hv	Submerged	N
Scrophulariaceae	*Limnophila indica*	S_Li/E_Li	Submerged/Emergent	Y
Scrophulariaceae	*Limnophila sessiliflora*	E_Ls	Submerged	Y
Onagraceae	*Ludwigia ovalis*	E_Lo	Submerged	N
Onagraceae	*Ludwigia peploides* subsp. *stipulacea*	E_Lp	Submerged	N
Haloragidaceae	*Myriophyllum aquaticum*	S_Ma/E_Ma	Submerged/Emergent	N
Haloragidaceae	*Myriophyllum propinquum*	S_Mp	Emergent	N

Haloragidaceae	*Myriophyllum spicatum*	S_Ms	Submerged	N
Haloragidaceae	*Myriophyllum verticicillatum*	S_Mv	Submerged	N
Hydrocharitaceae	*Najas guadalupensis*	S_Ng	Submerged	Y
Hydrocharitaceae	*Vallisneria denseserrulata*	S_Vd	Submerged	Y
Potamogetonaceae	*Potamogeton lucens*	S_Pl	Submerged	Y
Potamogetonaceae	*Potamogeton maackianus*	S_Pm	Submerged	Y
Potamogetonaceae	*Potamogeton octandrus*	S_Po	Submerged	Y
Potamogetonaceae	*Potamogeton perfoliatus*	S_Pp	Submerged	Y
Potamogetonaceae	*Potamogeton wrightii*	S_Pw/E_Pw	Submerged/Emergent	Y
Lythraceae	*Rotala rotundifolia*	S_Rr/E_Rr	Submerged/Emergent	N

Table S2. Statistical summary of the differences in periphyton Chla and AFDW between the two depths using Mann Whitney U test (n=3-5). Detailed species names are listed in Table S1. "/" means no results.

**Species**	**Periphyton Chla**		**AFDW**	
	**W**	***p* Value**	**W**	***p* Value**
E_Hy	4	0.77	2	0.77
E_Li	0	0.22	0	0.22
E_Lo	0	0.25	0	0.22
E_Lp	2	0.77	0	0.15
E_Ma	3	0.66	0	0.15
E_Mp	0	0.11	/	/
E_Pw	9	0.08	1	0.19
S_Cc	4	0.77	6	0.15
S_Hs	6	0.11	/	/
S_Hv	9	0.38	6	0.66
S_Li	5	0.86	6	0.49
S_Ma	9	0.06	6	0.11
S_Ng	7	0.89	5	0.47
S_Pl	9	0.90	4	0.18
S_Pm	0	0.08	0	0.14
S_Pp	0	0.06	0	0.06
S_Pw	3	0.82	1	0.25
S_Rr	4	1.00	0	0.22
S_Vd	2	1.00	2	1.00

Table S3. Statistical summary of the differences in ramet number and rhizome length between the two depths using Mann Whitney U test (n=3-5). Species names are listed in Table S1.

Species	Ramet number		Rhizome length	
	W	*p* Value	W	*p* Value
S_Vd	4.5	0.22	3	1.00
S_Pw	5.5	0.47	5.5	0.48
S_Pm	3	0.32	3	0.44
S_Pl	7.5	0.88	12	0.25
E_Pw	5.5	0.65	4	0.83
E_Hy	3	0.32	2	1.00

Table S4. Correlation analysis between periphyton biomass and macrophyte biomass. 'E' refers to emergent macrophytes and 'S' to submerged macrophytes. Two depths (50 cm and 100 cm) are indicated by 50 and 100.

Biomass	E_50	S_50	E_100	S_100
Periphyton Chla	$p < 0.05$, $R^2 = 0.37$	$p < 0.01$, $R^2 = 0.26$	$p > 0.05$	$p = 0.01$, $R^2 = 0.16$
Periphyton AFDW	$p < 0.01$, $R^2 = 0.64$	$p < 0.001$, $R^2 = 0.47$	$p > 0.05$	p < 0.001, $R^2 = 0.63$

The biomass of emergent macrophyte species and periphyton biomass did not correlate significantly at 100 cm depth, whereas plant biomass was negatively correlated with periphyton Chla and AFDW at 50 cm. For submerged macrophytes, periphyton Chla and AFDW were negatively correlated with periphyton biomass independently of water depth (Table S4).
